# Complete Genome Sequence of Community-Associated Methicillin-Resistant Staphylococcus aureus Sequence Type 1, SCC*mec* IV[2B], Isolated in the 1990s from Northern Western Australia

**DOI:** 10.1128/MRA.00796-21

**Published:** 2021-09-16

**Authors:** Nicola M. Karakatsanis, Shakeel Mowlaboccus, Elena Colombi, Julie C. Pearson, Joshua P. Ramsay, Geoffrey W. Coombs

**Affiliations:** a Curtin Health Innovation Research Institute, Curtin University, Perth, Western Australia, Australia; b Curtin Medical School, Curtin University, Perth, Western Australia, Australia; c College of Science, Health, Engineering and Education, Murdoch University, Murdoch, Western Australia, Australia; d Department of Microbiology, PathWest Laboratory Medicine WA, Fiona Stanley Hospital, Murdoch, Western Australia, Australia; University of Rochester School of Medicine and Dentistry

## Abstract

Sequence type 1 (ST1) methicillin-resistant Staphylococcus aureus (MRSA) SCC*mec* IV[2B] has become one of the most common community-associated MRSA clones in Australia. We report the complete genome sequence of one of the earliest isolated Australian S. aureus ST1-MRSA-IV strains, WBG8287, isolated from an Indigenous Australian patient living in the remote Kimberley region of Western Australia.

## ANNOUNCEMENT

Community-associated methicillin-resistant Staphylococcus aureus (CA-MRSA) first emerged in Australia in the 1980s (1, 2). An early CA-MRSA strain identified in Australia was WBG8287, a sequence type 1 (ST1) MRSA-IV[2B] strain isolated from an Indigenous patient from a remote community in the Kimberley region of Western Australia (WA) (1, 3). Colloquially known as WA-1 MRSA, ST1-MRSA-IV[2B] has become one of the most common CA-MRSA clones in Australia (1). Here, we present the complete genome sequence of WBG8287 to provide insight into the emergence and epidemiology of CA-MRSA in WA and provide a high-quality reference genome sequence for the WA-1-MRSA strains in current circulation.

S. aureus WBG8287, isolated as previously described (3), was rehydrated from a lyophilized stock in tryptic soy broth (TSB) and grown overnight on blood agar at 37°C. WBG8287 was grown overnight in 5 ml TSB at 37°C, and cells were harvested by centrifugation. The cells were resuspended in 200 μl Tris-EDTA (TE) buffer containing 1 mg/ml RNase (Sigma-Aldrich, USA) and 100 μg/ml lysostaphin (Sigma-Aldrich, USA) and incubated at 37°C for 10 min. For Oxford Nanopore Technologies (ONT) MinION sequencing, DNA was extracted using the FavorPrep FABGK-001-2 kit (Favorgen, Australia). DNA from a separate culture was extracted using Qiagen kit number 69506 (Germany) for Illumina NextSeq sequencing. No DNA shearing or size selection was carried out.

The WBG8287 genome was sequenced using the ONT SQK-RAD004 kit, the ONT MinION R.9.4.1 FLO-MIN106 flow cell, and the MinION Mk1B (ONT, UK), using MinKNOW v20.10.3 and MinKNOW Core v4.1.2 software. Base calling was performed using Guppy v4.4.1 + 1c81d62 (ONT) with the dna_r9.4.1_450bps_hac.cfg model. Sequencing produced 470,277 reads and a read-length *N*_50_ value of 2,525 bases. The unfiltered MinION reads were assembled using Flye v2.8.3-b1695 (4), which produced three circular contigs. The assembly was polished with the MinION reads 6× using Minimap2 v2.17-r941 (5) and Racon v1.4.15 (https://github.com/lbcb-sci/racon). WBG8287 was additionally sequenced using the Illumina NextSeq 500 platform, the Nextera XT DNA library preparation kit (paired-end format) (Illumina, USA), and the NextSeq 500/550 kit v2.5 (300-cycle format) (Illumina). The Illumina sequencing produced 2,083,742 reads, with a maximum read length of 151 bp and a mean read length of 147 bp. The Nesoni clip tool (github.com/Victorian-Bioinformatics-Consortium/nesoni) was used to remove adaptor sequences and quality filter reads. The assembly was polished with the Illumina reads 5× using Minimap2 v2.17-r941 (5) and Pilon v1.23 (6). Circlator v1.5.5 (7) was used to set the chromosome start position, and coverage statistics were generated using Qualimap v2.2.2-dev (8). The genome assembly of WBG8287 produced a 2,779,154 bp chromosome and plasmids pWBG750 (19,830 bp) and pWBG751 (2,473 bp) with 32% average GC content. The MinION reads mapped to the genome with 220-fold coverage, and the Illumina reads mapped with 109-fold coverage. Genome annotation was performed using the NCBI Prokaryotic Genome Annotation Pipeline v5.1 (9). Default parameters were used for all software unless otherwise specified.

### Data availability.

The WBG8287 sequences are deposited in NCBI GenBank under accession numbers CP070986.1 (chromosome), CP070987.1 (pWBG750), and CP070988.1 (pWBG751). The sequencing reads have been deposited in the Sequence Read Archive under accession numbers SRX11246054 and SRX11246053.
